# Characterization of the Microbiome of Corals with Stony Coral Tissue Loss Disease along Florida’s Coral Reef

**DOI:** 10.3390/microorganisms9112181

**Published:** 2021-10-20

**Authors:** Abigail S. Clark, Sara D. Williams, Kerry Maxwell, Stephanie M. Rosales, Lindsay K. Huebner, Jan H. Landsberg, John H. Hunt, Erinn M. Muller

**Affiliations:** 1 Elizabeth Moore International Center for Coral Reef Research and Restoration, Mote Marine Laboratory, Summerland Key, FL 33042, USA; emuller@mote.org; 2 Mote Marine Laboratory, Sarasota, FL 34236, USA; swilliams@mote.org; 3 South Florida Regional Laboratory, Fish & Wildlife Research Institute, Florida Fish & Wildlife Conservation Commission, Marathon, FL 33050, USA; Kerry.Maxwell@myfwc.com (K.M.); John.Hunt@myfwc.com (J.H.H.); 4 Cooperative Institute for Marine and Atmospheric Studies, University of Miami, Miami, FL 33149, USA; Stephanie.Rosales@noaa.gov; 5 Atlantic Oceanographic and Meteorological Laboratory, National Oceanic and Atmospheric Administration, Miami, FL 33149, USA; 6 Fish & Wildlife Research Institute, Florida Fish & Wildlife Conservation Commission, Saint Petersburg, FL 33701, USA; Lindsay.Huebner@myfwc.com (L.K.H.); Jan.Landsberg@myfwc.com (J.H.L.)

**Keywords:** stony coral tissue loss disease, SCTLD, coral disease, bacteria, microbiome, Rhodobacterales, Clostridiales

## Abstract

Stony coral tissue loss disease (SCTLD) is an emergent and often lethal coral disease that was first reported near Miami, FL (USA) in 2014. Our objective was to determine if coral colonies showing signs of SCTLD possess a specific microbial signature across five susceptible species sampled in Florida’s Coral Reef. Three sample types were collected: lesion tissue and apparently unaffected tissue of diseased colonies, and tissue of apparently healthy colonies. Using 16S rRNA high-throughput gene sequencing, our results show that, for every species, the microbial community composition of lesion tissue was significantly different from healthy colony tissue and from the unaffected tissue of diseased colonies. The lesion tissue of all but one species (*Siderastrea siderea*) had higher relative abundances of the order Rhodobacterales compared with other types of tissue samples, which may partly explain why *S. siderea* lesions often differed in appearance compared to other species. The order Clostridiales was also present at relatively high abundances in the lesion tissue of three species compared to healthy and unaffected tissues. Stress often leads to the dysbiosis of coral microbiomes and increases the abundance of opportunistic pathogens. The present study suggests that Rhodobacterales and Clostridiales likely play an important role in SCTLD.

## 1. Introduction

Corals host a diversity of microorganisms composed of viruses, fungi, archaea, endolithic algae, protozoa, bacteria, and algal symbionts [1,2,3,4,5]. Microbes living in or on a coral can be beneficial to the coral host by playing important roles in the cycling and recycling of nutrients [6,7,8], the production of amino acids [8,9], protection against pathogens [10,11], and enhancing larval settlement and metamorphosis [12]. Disturbances, such as those caused by climate change, can alter coral–microbe interactions and lead to bleaching, disease, and the mortality of the coral host [13,14,15].

Climate change, overfishing, and pollution are among the many stressors contributing to the decline of coral reef health worldwide [16,17]. As a result of these stressors and others (e.g., sedimentation), there has been an increase in widespread bleaching events, disease incidence, and subsequent mortality among coral communities in recent decades [18,19,20,21,22]. In 2014, a novel coral disease was documented off Southeast Florida, USA, and has since spread through Florida’s Coral Reef (FCR) and across much of the Caribbean region [23,24]. Stony coral tissue loss disease (SCTLD) is believed to affect more than 23 species of scleractinian corals [25]. Signs of active SCTLD include focal or multifocal lesions moving at chronic to acute rates, followed by partial or whole colony tissue loss, often resulting in complete mortality of coral colonies [23,26,27,28,29].

While much remains unknown about SCTLD, advances in our understanding of the etiology [30], spatial epidemiology [31,32,33,34], diagnostics [35], and treatment [29,36,37] of SCTLD have been achieved. Corals, especially many of the major Caribbean reef-building species, exposed to SCTLD experience high mortality [23,38]. For example, >97% of *Meandrina meandrites* and *Dichocoenia stokesii* colonies monitored off Miami-Dade County, FL, USA, died in the year following the onset of the SCTLD outbreak [23]. SCTLD is presumed to spread through waterborne transmission and by direct contact [26], and barotropic oceanic currents correlate with the spatio-temporal progress of the disease throughout FCR [31]. The spatio-temporal dynamics indicate that SCTLD follows a contagion model over both large [32] and small [34] spatial scales, again potentially indicating a novel pathogen driving disease dynamics. A causative agent of SCTLD has not yet been identified, making it challenging to identify possible vectors or intermediate hosts. However, a recent study isolated *Vibrio coralliilyticus* from some active SCTLD lesions and revealed that *V. coralliilyticus* may play an opportunistic role in exacerbating the disease [35]. Thus, bacteria play an important, possibly secondary, role in lesion advancement in SCTLD [30], and identifying the microbes associated with SCTLD lesions is critical for identifying the key microbiota likely involved in disease progression.

Recent studies have used high-throughput 16S rRNA sequencing to describe the microbiomes of active SCTLD lesions. Meyer et al. [39] compared the microbial community compositions of four species of coral, each displaying signs of active SCTLD: *Montastraea cavernosa*, *Orbicella faveolata*, *Diploria labyrinthiformis*, and *Dichocoenia stokesii*. The following bacterial orders were enriched in SCTLD lesions of all but one coral species (*O. faveolata*): Flavobacteriales, Clostridiales, Rhodobacterales, Alteromonadales, and Vibrionales. Rosales et al. [40] identified two bacterial orders, Rhodobacterales and Rhizobiales, in the active lesions of *Stephanocoenia intersepta*, *Diploria labyrinthiformis*, *Dichocoenia stokesii*, and *Meandrina meandrites* that were more prevalent compared to the microbiomes of the tissue of apparently healthy colonies and the unaffected tissues of diseased colonies. Water samples collected at sites with SCTLD also had relatively high abundances of Rhodobacterales compared to sites with no signs of SCTLD, and both water and sediments shared sequences with lesions of diseased corals [40].

The objectives of the present study were to determine if a specific microbial signature exists in the lesions of corals experiencing SCTLD by characterizing the microbial community (1) between disease outbreak zones (epidemic and vulnerable zones), (2) among sites within each zone, and (3) among coral sample tissue types (lesion and unaffected tissues from diseased colonies, and tissue from apparently healthy colonies). To evaluate the microbial diversity and composition of corals affected by SCTLD, tissues were collected from five coral species; *Colpophyllia natans*, *Pseudodiploria strigosa*, *Montastraea cavernosa*, *O. faveolata*, and *Siderastrea siderea*. Samples were collected from sites in the Middle Florida Keys (the epidemic zone) and the Lower Florida Keys (the vulnerable zone). The SCTLD microbial signatures of *M. cavernosa* and *O. faveolata* have been characterized previously for samples collected from the endemic zone in Southeast Florida [39], allowing for species-specific comparisons across FCR. Additionally, we collected water samples from our study sites, allowing for a comparison of our results to a previous study which examined the SCTLD microbial signatures in water [40].

## 2. Materials and Methods

### 2.1. Sample Collection

**Figure 1:**
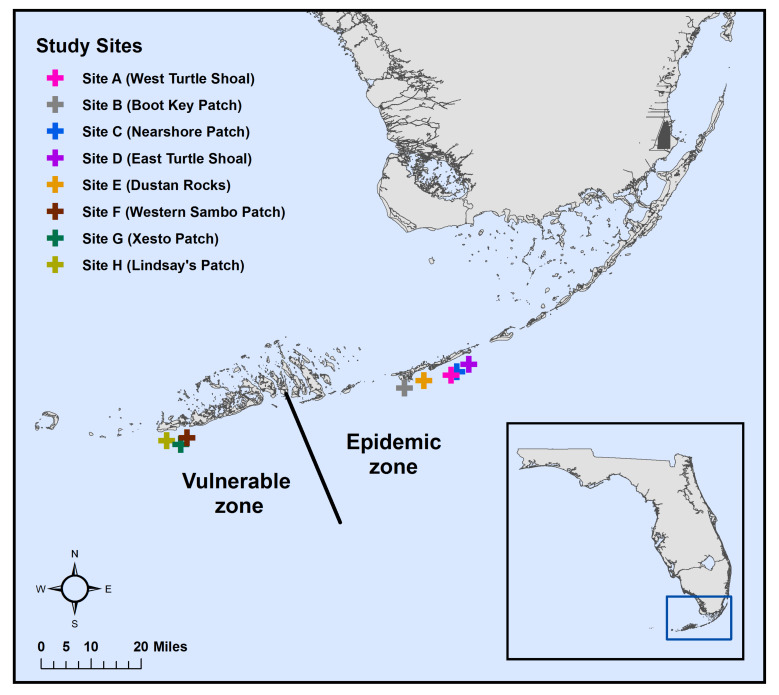
Map of sampling sites (n = 8; represented by symbols) along Florida’s Coral Reef (FCR). Five sites (Sites A–E) were located in the epidemic zone, an area of FCR where stony coral tissue loss disease (SCTLD) was active at the time. Three sites (Sites F–H) were located ahead of the epidemic zone in an area designated the vulnerable zone. The black line indicates the approximate front of SCTLD at the time of sampling in 2018. Coral reef names corresponding to Sites A–H are in parentheses.

From April to June 2018, coral tissue (n = 363) and water samples (n = 24) were collected along Florida’s Coral Reef (FCR) in the Florida Keys, USA (Figure 1; Table 1). Samples were collected at eight sites: five sites in the epidemic zone (Sites A–E), the area of FCR with an active stony coral tissue loss disease (SCTLD) outbreak, and three sites in the vulnerable zone (Sites F–H), the area of FCR located ahead of the epidemic zone. Five coral species susceptible to SCTLD [25] were sampled: *Colpophyllia natans*, *Pseudodiploria strigosa*, *Montastraea cavernosa*, *Orbicella faveolata*, and *Siderastrea siderea*. The colonies sampled here were also sampled for histopathological analysis using light microscopy and transmission electron microscopy [30,41].

**Figure 2:**
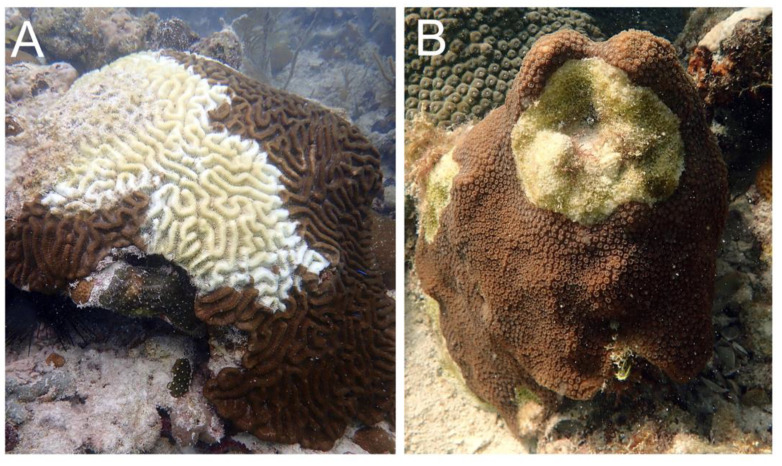
Representative photos of colonies with stony coral tissue loss disease. (**A**) *Colpophyllia natans* with acute tissue loss and (**B**) *Orbicella faveolata* with chronic tissue loss.

Techniques and methods developed previously [40] were used to collect coral tissue/mucus and water samples at each of the sites by divers on SCUBA. Divers used a sterile, 10 mL syringe to scrape and remove tissue from most colonies, except *S. siderea*. Because syringe tips would frequently break when attempting to sample *S. siderea* colonies, divers used a sterile corer to scrape off the tissue while simultaneously using a syringe to capture the scrapings and mucus. In the epidemic zone, three types of coral samples were collected per species: lesion tissue of diseased colonies (DL; along the disease margin of active lesions; Figure 2), unaffected tissue of diseased colonies (DU; the areas not showing obvious external signs of SCTLD on visibly diseased colonies), and tissue of apparently healthy colonies (AH; colonies with no external signs of SCTLD). For each diseased colony, the DU tissue sample was collected before the DL tissue sample to minimize the risk of contamination from sampling the lesion tissue. At every epidemic site, divers collected DL and DU tissue samples from three to five colonies per species and AH tissue samples from three colonies per species (Table 1). At the three sites in the vulnerable zone, AH tissue samples were collected from two to three colonies per species. Additionally, at each site in each zone, three water samples were collected by inverting sterile, 1 L bottles approximately 0.5 m above the benthos, in proximity of the coral colony sampling area.

On the boat, coral tissue samples were transferred from syringes into plastic tubes before being transported, along with the water sample bottles, on ice directly from the sampling sites to the South Florida Regional Laboratory of the Florida Fish & Wildlife Conservation Commission’s Fish and Wildlife Research Institute (FWC-FWRI, Marathon, FL, USA). At FWRI, water samples were filtered through 0.2 µm filters. All water filters and coral tissue samples were flash-frozen using liquid nitrogen before being immediately transferred to −80 °C for storage.

### 2.2. Sample Processing

DNA was isolated from all samples using DNeasy PowerSoil Kits (QIAGEN, Germantown, MD, USA) and modifications to the manufacturer’s protocol. The protocol optimized and used previously [40] was employed to isolate DNA from all samples in the present study. However, to standardize concentrations of DNA isolated from the five coral species, the amount of starting material processed for DNA varied depending on the coral species. For *S. siderea*, DNA was isolated from 6 mL of the coral tissue/mucus slurries, but 2 mL of samples were used for the other four species. Each slurry was then centrifuged and the supernatant was discarded so that only a pellet remained in each tube. For the water samples, half of every 0.2 µm filter was cut into small pieces, which were then transferred to their respective tubes. Next, phenol: chloroform: isoamyl alcohol (pH 7–8; Fisher Scientific Company LLC, Hanover Park, IL, USA) and Solution C1 of the DNeasy PowerSoil Kit were added to every tube, vortexed for 10 min, and centrifuged. The supernatants were then transferred to new tubes to which Solutions C2 and C3 of the DNeasy PowerSoil Kit were added. The process of adding reagents and centrifuging samples continued, as described previously [40], until left with 60 µL of isolated DNA. DNA concentrations and quality were measured with a NanoDrop One^TM^ Microvolume UV-Vis Spectrophotometer (Thermo Fisher Scientific, Waltham, MA, USA).

DNA was then processed using high-throughput 16S rRNA sequencing (www.mrdnalab.com (accessed on 12 October 2021), Shallowater, TX, USA). The 16S rRNA gene variable region (V4) was amplified using PCR primers 515F (GTGCCAGCMGCCGCGGTAA; Original Earth Microbiome Project) [42] and 806R (GGACTACVSGGGTATCTAAT; Archaea 806R) [43], 1 µL of DNA, and the HotStarTaq Plus Kit (QIAGEN, Germantown, MD, USA). The following thermocycler conditions were used: 94 °C for 3 min, 30 cycles of 94 °C each for 30 s, 53 °C for 40 s, 72 °C for 1 min, and a final elongation step at 72 °C for 5 min. After PCR products were visualized on a 2% agarose gel, samples were pooled and, subsequently, purified using AMPure XP beads (Beckman Coulter Life Sciences, Indianapolis, IL, USA). The pooled sample was processed with a MiSeq Reagent Kit v3 and paired-end sequenced using two lanes of a MiSeq (Illumina, San Diego, CA, USA).

### 2.3. Data Analysis

Sequences were demultiplexed using the MR DNA free software application, FASTqProcessor (version 20.03.02; www.mrdnalab.com (accessed on 12 October 2021), Shallowater, TX, USA). All other data analyses were completed in the program R (version 4.0.5; R Foundation for Statistical Computing, Vienna, Austria) [44]. The DADA2 pipeline [45] was used to determine amplicon sequence variants (ASVs) by checking read quality, filtering and trimming sequences, dereplicating, merging sequences, and removing chimeras. Since samples were sequenced on two lanes of an Illumina MiSeq, each lane was processed through the DADA2 pipeline individually. Taxonomy was assigned using the Silva (version 132; European Organization for Nuclear Research, Geneva, Switzerland) [46] reference database. Taxonomy tables, ASV tables, and sample data from the two sequencing lanes were then merged and further analysis and processing was completed using the phyloseq package [47]. Following taxonomic assignment, sequences from the coral host, algal symbionts, and eukaryotes were removed. The resulting ASV count data were filtered so that only ASVs present in a minimum of four samples were included in the analysis (similar to Rosales et al. [40]). ASV count data were then normalized using a centered log-ratio (CLR) transformation.

Differences in microbial communities were assessed between zone (epidemic vs. vulnerable), sites within each zone, and tissue sample type (DL, DU, and AH) for each coral species. All tissue sample types (DL, DU, and AH) collected in the epidemic zone were combined for each coral species when comparing sites within the epidemic zone. To measure beta diversity differences, the vegdist function of the R package ‘vegan’ [48] and the CLR-transformed data were used to calculate dissimilarity indices. These indices were then tested for the homogeneity of groups dispersion using the betadisper function and were, subsequently, tested for significance using a permutation test (Permutest) and a Tukey’s honest significant difference (HSD) test. Next, permutational multivariate analysis of variance (PERMANOVA) and pairwise PERMANOVA tests were used to measure differences in bacterial communities among zones, sites, and sample types for each coral species. All PERMANOVA tests were performed using the adonis function in the R package ‘vegan’ with a Euclidean distance. Next, a pairwise comparison was conducted and the *p*-values generated from this comparison were adjusted using a Bonferroni correction [49]. Non-metric multidimensional scaling (NMDS) with a Euclidean distance was used to generate ordination plots and further evaluate the bacterial community differences among samples.

Alpha diversity metrics (species richness and Shannon diversity) were analyzed using the ‘vegan’ package. Shapiro–Wilks tests were used to test for normality conditions. If the data met normality assumptions, a one-way analysis of variance (ANOVA) and Tukey’s post-hoc test were used. If the data did not meet normality assumptions, then a Kruskal–Wallis rank sum test was used. When significant differences were detected, a post-hoc Wilcoxon signed rank test was then used to determine which groups were significantly different.

An analysis of composition of microbiomes (ANCOM) was used to identify significant ASVs that were differentially abundant among the tissue sample types for each coral species. To perform the ANCOM, a phyloseq object containing the ASV count data and taxonomy was used [50]. The ANCOM results were considered significant if the calculated W-statistic exceeded a detection threshold of 0.7. For each coral species, ANCOM was run with tissue sample type as the independent variable tested and, for water samples, disease outbreak zone was the independent variable. Bar plots displaying the mean relative abundance of bacterial classes present in each species were generated.

## 3. Results

**Figure 3:**
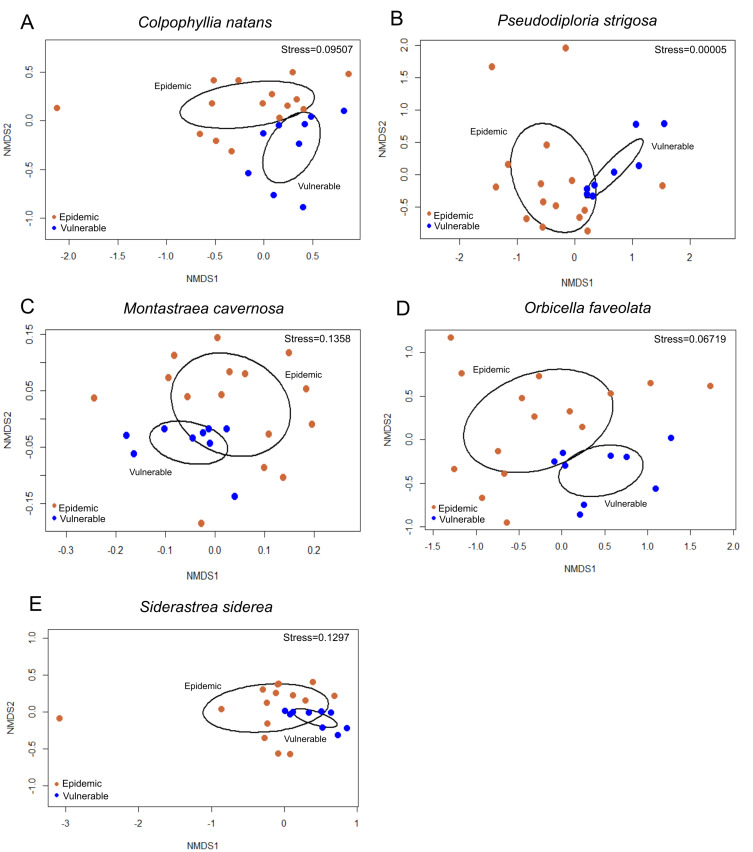
Non-metric Multidimensional Scaling (NMDS) ordination plots of amplicon sequence variants (ASVs) in the tissues of apparently healthy colonies of five coral species: (**A**) *Colpophyllia natans*, (**B**) *Pseudodiploria strigosa*, (**C**) *Montastraea cavernosa*, (**D**) *Orbicella faveolata*, and (**E**) *Siderastrea siderea*. Tissues of apparently healthy colonies were collected from the epidemic zone (orange) and the vulnerable zone (blue) of stony coral tissue loss disease. NMDS was performed using a Euclidean distance.

After data filtration, 16,876 ASVs remained and were included in the analysis. A permutational multivariate analysis of variance (PERMANOVA) test found that bacterial communities of tissue sampled from apparently healthy (AH) colonies in the vulnerable zone were significantly different from the bacterial communities associated with AH tissue in the epidemic zone for each species: *Colpophyllia natans* (Figure 3A; F_1,16_ = 1.6007, R^2^ = 0.06298, *p* = 0.0053), *Pseudodiploria strigosa* (Figure 3B; F_1,15_ = 2.6, R^2^ = 0.1044, *p* < 0.0001), *Montastraea cavernosa* (Figure 3C; F_1,15_ = 1.7394, R^2^ = 0.07, *p* = 0.0002), *Orbicella faveolata* (Figure 3D; F_1,15_ = 2.4137, R^2^ = 0.09132, *p* < 0.0001), and *Siderastrea siderea* (Figure 3E; F_1,15_ = 1.935, R^2^ = 0.07603, *p* < 0.0001). Furthermore, there were also significant differences in the bacterial community of the coral species among sites within the zones: *C. natans* (F_6,16_ = 1.3024, R^2^ = 0.3075, *p* < 0.0001), *P. strigosa* (F_6,15_ = 1.2162, R^2^ = 0.2931, *p* = 0.0093), *M. cavernosa* (F_6,16_ = 1.1849, R^2^ = 0.2861, *p* = 0.0003), *O. faveolata* (F_6,16_ = 1.3363, R^2^ = 0.3034, *p* = 0.0002), and *S. siderea* (F_6,16_ = 1.2528, R^2^ = 0.2953, *p* < 0.0001). As a result of the differences across zones and among sites, the ASVs generated from colonies in the vulnerable zone were analyzed independently of ASVs associated with the epidemic zone.

### 3.1. Microbial Community Analysis

#### 3.1.1. Microbial Communities among Sample Types

**Figure 4:**
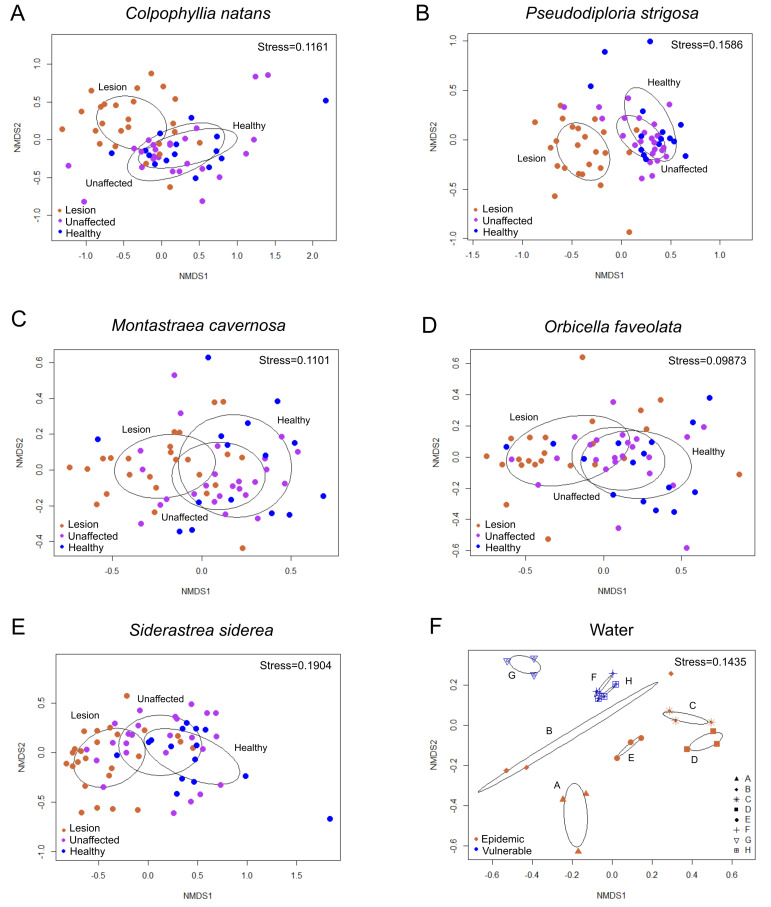
Non-metric Multidimensional Scaling (NMDS) ordination plots of amplicon sequence variants (ASVs) in lesion tissue (orange) and unaffected tissue (purple) of colonies with stony coral tissue loss disease, and tissue of apparently healthy colonies (blue) of five coral species: (**A**) *Colpophyllia natans*, (**B**) *Pseudodiploria strigosa*, (**C**) *Montastraea cavernosa*, (**D**) *Orbicella faveolata*, and (**E**) *Siderastrea siderea*. These data reflect samples collected in the epidemic zone. NMDS ordination plots of ASVs in (**F**) water samples reflect samples collected in the epidemic zone (Sites A–E; orange) and vulnerable zone (Sites F–H; blue). NMDS was performed using a Euclidean distance.

There were significant differences in the bacterial communities of lesion tissue (DL) from diseased colonies compared with unaffected tissue (DU) from diseased colonies and AH tissues for every species in the epidemic zone: *C. natans* (Figure 4A; F_2,50_ = 1.797, R^2^ = 0.05, *p* = 0.0002), *P. strigosa* (Figure 4B; F_2,48_ = 2.0253, R^2^ = 0.061, *p* < 0.00001), *M. cavernosa* (Figure 4C; F_2,50_ = 1.6764, R^2^ = 0.05, *p* = 0.0005), *O. faveolata* (Figure 4D; F_2,46_ = 1.5014, R^2^ = 0.048, *p* = 0.0018), and *S. siderea* (Figure 4E; F_2,50_ = 1.7955, R^2^ = 0.05, *p* < 0.00001). A pairwise comparison of the three tissue sample types revealed that DL tissue was significantly different compared to DU and AH tissues of *C. natans*, *P. strigosa*, *S. siderea* (adjusted *p*-value (*p_adj*) = 0.0030 for both comparisons for all three coral species), *O. faveolata* (*p_adj* = 0.0060 and *p_adj* = 0.012 for DU and AH tissues, respectively), and *M. cavernosa* (*p_adj* = 0.0030 and *p_adj* = 0.015 for DU and AH tissues, respectively).

#### 3.1.2. Microbial Communities among Sites within Zones

Among sites in the epidemic zone, there were significant differences in the microbial communities of all three tissue sample types (DL, DU, and AH) pooled (Table S1). However, there were no significant differences in the microbial communities of AH tissues among sites in the vulnerable zone. There were significant differences in the bacterial communities of water collected in the vulnerable zone compared to the epidemic zone (Figure 4F; F_1,16_ = 3.3523, R^2^ = 0.11, *p* = 0.001).

### 3.2. Beta Diversity Analysis

#### 3.2.1. Beta Diversity between Vulnerable and Epidemic Zones

Microbial beta diversity as measured by the betadisper analysis did not significantly differ between the epidemic zone (all three tissue sample types pooled) and the vulnerable zone (AH tissue only) for each coral species: *C. natans* (F_1,72_ = 1.6604, *p* = 0.21), *P. strigosa* (F_1,69_ = 2.7863, *p* = 0.11), *M. cavernosa* (F_1,72_ = 0.5917, *p* = 0.46), and *O. faveolata* (F_1,68_ = 0.2523, *p* = 0.62), except for *S. siderea* (F_1,72_ = 4.0684, *p* = 0.038). Specifically, *S. siderea* in the epidemic zone was more diverse compared with the vulnerable zone (*p_adj* = 0.047). There were also no significant differences in the beta diversity of water collected in the epidemic zone compared to the vulnerable zone (F_1,22_ = 3.0978, *p* = 0.091).

#### 3.2.2. Beta Diversity among Sites within Zones

There were no significant differences in dispersion among sites within the epidemic zone or within the vulnerable zone for each species: *C. natans* (epidemic: F_4,60_ = 1.9158, *p* = 0.11; vulnerable: F_2,6_ = 0.2108, *p* = 0.74), *P. strigosa* (epidemic: F_4,58_ = 1.1991, *p* = 0.32; vulnerable: F_2,5_ = 0.1423, *p* = 0.93), *M. cavernosa* (epidemic: F_4,60_ = 2.0625, *p* = 0.10; vulnerable: F_2,6_ = 0.4821, *p* = 0.65), *O. faveolata* (epidemic: F_4,56_ = 0.4489, *p* = 0.77; vulnerable: F_2,6_ = 4.653, *p* = 0.089), and *S. siderea* (epidemic: F_4,60_ = 0.4481, *p* = 0.76; vulnerable: F_2,6_ = 0.1885, *p* = 0.92). There were no significant differences in the beta diversity of water samples among sites in the epidemic zone (F_4,10_ = 3.2959, *p* = 0.066) nor the vulnerable zone (F_2,6_ = 2.8082, *p* = 0.14).

#### 3.2.3. Beta Diversity among Coral Tissue Sample Types in the Epidemic Zone

There were significant differences in the dispersion of microbial communities (i.e., beta diversity) among coral tissue sample types (DL, DU, and AH) in the epidemic zone for four species: *C. natans* (F_2,62_ = 4.7286, *p* = 0.0070), *M. cavernosa* (F_2,62_ = 9.1288, *p* = 0.0010), *O. faveolata* (F_2,58_ = 5.793, *p* = 0.0030), and *S. siderea* (F_2,62_ = 14.051, *p* = 0.0010; Figure 4A,C–E). Pairwise comparisons revealed that DL tissue of *C. natans* was more dispersed in only AH tissue (*p_adj* = 0.013) but not DU tissue. However, DL tissue was more dispersed than both DU and AH tissues for *M. cavernosa* (*p_adj* = 0.0011 and *p_adj* = 0.0029, respectively), *O. faveolata* (*p_adj* = 0.027 and 0.0087, respectively), and *S. siderea* (*p_adj* = 0.00095 and *p_adj* = 0.00002, respectively). There were no significant differences in dispersion among *P. strigosa* tissue sample types (F_2,60_ = 1.6297, *p* = 0.20; Figure 4B).

#### 3.2.4. Beta Diversity of Apparently Healthy Colony Tissue in Vulnerable and Epidemic Zones

There were no significant differences in the dispersion of microbial communities (i.e., beta diversity) of AH tissue between the vulnerable zone and the epidemic zone for four species: *C. natans* (F_1,22_ = 0.0543, *p* = 0.80), *M. cavernosa* (F_1,22_ = 0.2821, *p* = 0.63), *O. faveolata* (F_1,22_ = 0.5641, *p* = 0.47), and *S. siderea* (F_1,22_ = 0.306, *p* = 0.55). However, the AH tissue of *P. strigosa* colonies in the vulnerable zone had a greater dispersion of microbial communities than of those in the epidemic zone (F_1,21_ = 5.5737, *p_adj* = 0.028).

### 3.3. Alpha Diversity Analysis

#### 3.3.1. Alpha Diversity between Vulnerable and Epidemic Zones

There were no significant differences in alpha diversity of the bacterial community between the epidemic zone (all three tissue sample types pooled) and the vulnerable zone (AH tissue only) for each coral species: *C. natans* (species richness: F_1,72_ = 0.063, *p* = 0.80; Shannon diversity: F_1,68_ = 1.477, *p* = 0.23; Figure S1A), *M. cavernosa* (species richness: F_1,72_ = 0.936, *p* = 0.34; Shannon diversity: F_1,72_ = 0.398, *p* = 0.53; Figure S1C), *O. faveolata* (species richness: F_1,68_ = 1.821, *p* = 0.18; Shannon diversity: F_1,68_ = 0.504, *p* = 0.48; Figure S1D), and *S. siderea* (species richness: F_1,72_ = 1.83, *p* = 0.18; Shannon diversity: F_1,72_ = 1.316, *p* = 0.26; Figure S1E), except for *P. strigosa* (species richness: F_1,69_ = 11.79, *p* = 0.0010; Shannon diversity: F_1,69_ = 6.589, *p* = 0.012; Figure S1B). *Pseudodiploria strigosa* samples collected in the vulnerable zone had a higher species richness and diversity than the epidemic zone. There were no differences in the species richness of water between zones (F_1,22_ = 3.093, *p* = 0.093; Figure S1F). However, significant differences were detected in the Shannon diversity (F_1,22_ = 8.337; *p* = 0.0086). Specifically, water samples collected in the vulnerable zone had a higher diversity than the epidemic zone.

#### 3.3.2. Alpha Diversity among Sites within Zones

There was only a significant difference in alpha diversity metrics among sites within the epidemic and vulnerable zones each for *C. natans* and *O. faveolata*. The species richness of *C. natans* was not significantly different among sites in the epidemic zone (F_4,60_ = 2.093, *p* = 0.093; Figure S2A), but there were differences in the Shannon diversity (F_4,60_ = 5.5, *p* = 0.0008). Dustan Rocks (Site E) was less diverse than Nearshore Patch (Site C) and East Turtle Shoal (Site D) (*p_adj* = 0.0032 and *p_adj* = 0.0069, respectively), and Nearshore Patch (Site C) had a higher diversity than West Turtle Shoal (Site A) (*p_adj* = 0.032). There were no significant differences in the alpha diversity of *C. natans* among sites in the vulnerable zone (species richness: F_2,6_ = 0.255, *p* = 0.78; Shannon diversity: F_2,6_ = 1.161, *p* = 0.37; Figure S3A). The species richness and diversity of *O. faveolata* were not significantly different among sites in the epidemic zone (species richness: F_4,56_ = 1.14, *p* = 0.35; Shannon diversity: F_4,56_ = 2.319, *p* = 0.068; Figure S2D). Furthermore, there were no differences in the Shannon diversity of *O. faveolata* among sites in the vulnerable zone (F_2,6_ = 1.185, *p* = 0.37; Figure S3D). However, there were significant differences among sites in the species richness of *O. faveolata* in the vulnerable zone (F_2,6_ = 5.978, *p* = 0.037). Species richness was higher in Western Sambo Patch (Site F) than Xesto Patch (Site G; *p_adj* = 0.039). For all other species, significant differences were not detected among sites in the vulnerable zone: *P. strigosa* (species richness: F_2,5_ = 0.61, *p* = 0.58; Shannon diversity: F_2,5_ = 0.146, *p* = 0.87; Figure S3B), *M. cavernosa* (species richness: F_2,6_ = 0.434, *p* = 0.67; Shannon diversity: F_2,6_ = 0.858, *p* = 0.47; Figure S3C), and *S. siderea* (species richness: F_2,6_ = 0.503, *p* = 0.63; Shannon diversity: F_2,6_ = 0.733, *p* = 0.52; Figure S3E). There were also no significant differences among sites in the epidemic zone for these species (*p* ≥ 0.1 for all comparisons, range: 0.10–96; Figure S2B,C,E).

There were no differences in the alpha diversity of water samples among sites in the epidemic zone (species richness: F_4,10_ = 0.854, *p* = 0.52; Shannon diversity: F_4,10_ = 2.816, *p* = 0.084), or for the species richness among sites in the vulnerable zone (F_2,6_ = 5.065, *p* = 0.052). However, there were Shannon diversity differences among sites in the vulnerable zone (F_2,6_ = 17.07, *p* = 0.0033). Xesto Patch (Site G) was more diverse than Western Sambo Patch (Site F) and Lindsay’s Patch (Site H) (*p_adj* = 0.011 and *p_adj* = 0.0035, respectively).

#### 3.3.3. Alpha Diversity among Coral Tissue Sample Types in the Epidemic Zone

**Figure 5:**
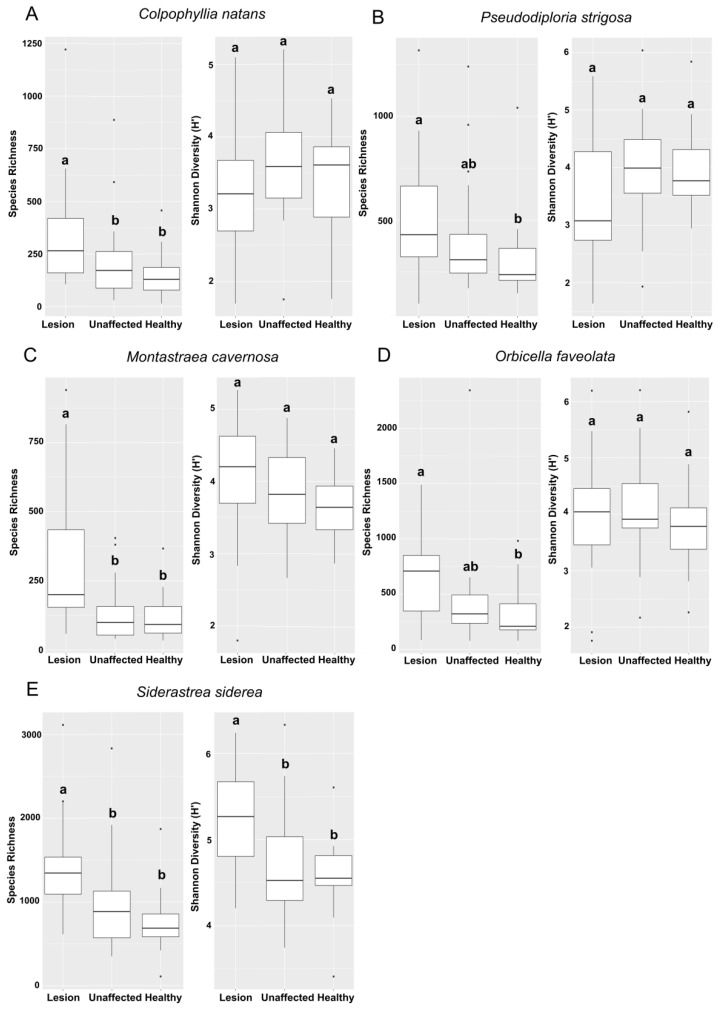
Microbial alpha diversity metrics (species richness and Shannon diversity) comparing lesion and unaffected tissues from colonies with stony coral tissue loss disease, and tissue of apparently healthy colonies, collected from five coral species: (**A**) *Colpophyllia natans*, (**B**) *Pseudodiploria strigosa*, (**C**) *Montastraea cavernosa*, (**D**) *Orbicella faveolata*, and (**E**) *Siderastrea siderea*. Letters denote significant differences determined by the post-hoc tests.

There were significant differences in the microbial species richness among the tissue sample types (DL, DU, and AH) of every coral species: *C. natans* (F_6,62_ = 6.605, *p* = 0.0025; Figure 5A), *P. strigosa* (X^2^ = 9.5497, df = 2, *p* = 0.0084; Figure 5B), *M. cavernosa* (F_2,62_ = 9.986, *p* = 0.00017; Figure 5C), *O. faveolata* (F_2,58_ = 5.435, *p* = 0.0069; Figure 5D), and *S. siderea* (F_2,62_ = 10.75, *p* < 0.00001; Figure 5E). DL tissue had a higher species richness than DU and AH tissues in *C. natans* (*p_adj* = 0.026 and *p_adj* = 0.0036, respectively), *M. cavernosa* (*p_adj* = 0.00058 and *p_adj* = 0.0019, respectively), and *S. siderea* (*p_adj* = 0.0046 and *p_adj* = 0.00014, respectively). DL tissue of *O. faveolata* and *P. strigosa* had a higher species richness than AH tissues only (*p_adj* = 0.0076 and *p_adj* = 0.018, respectively).

There were no significant differences in the Shannon species diversity among tissue sample types from *C. natans* (F_2,62_ = 1.311, *p* = 0.28; Figure 5A), *P. strigosa* (F_2,60_ = 3.065, *p* = 0.054; Figure 5B), *M. cavernosa* (F_2,62_ = 2.212, *p* = 0.12; Figure 5C), and *O. faveolata* (F_2,58_ = 0.488, *p* = 0.62; Figure 5D). However, there were significant differences in the Shannon species diversity of *S. siderea* tissues (F_2,62_ = 8.098, *p* = 0.00075; Figure 5E), with DL tissues more diverse than both DU and AH tissues for this species (*p_adj* = 0.0057 and *p_adj* = 0.0020, respectively).

#### 3.3.4. Alpha Diversity of Apparently Healthy Colony Tissue in Vulnerable and Epidemic Zones

**Figure 6:**
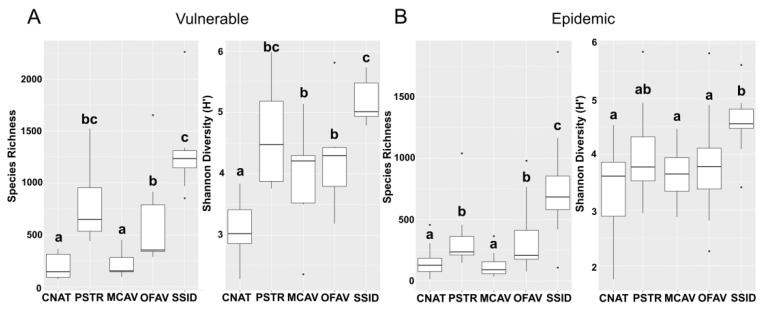
Microbial alpha diversity metrics (species richness and Shannon diversity) comparing tissue of apparently healthy colonies collected from five coral species, *Colpophyllia natans* (CNAT), *Pseudodiploria strigosa* (PSTR), *Montastraea cavernosa* (MCAV), *Orbicella faveolata* (OFAV), and *Siderastrea siderea* (SSID) in (**A**) the vulnerable zone and (**B**) the epidemic zone of stony coral tissue loss disease. Letters denote significant differences determined by the post-hoc tests.

For each coral species, there were significant differences in both microbial alpha diversity metrics of AH tissue in the vulnerable zone (species richness: F_4,39_ = 25.34, *p* < 0.00001; Shannon diversity: X^2^ = 11.92, df = 4, *p* < 0.00001; Figure 6A) and in the epidemic zone (species richness: X^2^ = 18.3, df = 4, *p* < 0.00001; Shannon diversity: X^2^ = 6.295, df = 4, *p* = 0.0002; Figure 6B). In the vulnerable zone, a pairwise comparison of microbial species richness between each coral species showed that *C. natans* and *M. cavernosa* each had a lower species richness compared to *O. faveolata*, *P. strigosa*, and *S. siderea* (*p_adj* < 0.001 for all comparisons, range: < 0.00001–0.0002). *Siderastrea siderea* had a higher species richness than *O. faveolata* (*p_adj* = 0.010). *Colpophyllia natans* had a lower Shannon diversity than the other four species: *M. cavernosa* (*p_adj* = 0.039), *O. faveolata* (*p_adj* = 0.0083), *P. strigosa* (*p_adj* = 0.00030), and *S. siderea* (*p_adj* < 0.00001). *Siderastrea siderea* was more diverse than *M. cavernosa* and *O. faveolata* (*p_adj* = 0.0065 and *p_adj* = 0.031, respectively).

As in the vulnerable zone, pairwise comparisons of AH tissue in the epidemic zone showed that *C. natans* and *M. cavernosa* had a lower species richness than *O. faveolata*, *P. strigosa*, and *S. siderea* (*p_adj* < 0.05 for all comparisons, range: < 0.00001–0.011; Figure 6B). Dissimilar to the vulnerable zone, *S. siderea* in the epidemic zone had a higher species richness than *P. strigosa* (*p_adj* = 0.0082) as well as *O. faveolata* (*p_adj* = 0.0029). *Siderastrea siderea* also had a higher Shannon diversity than *C. natans* (*p_adj* = 0.00010), *M. cavernosa* (*p_adj* = 0.0038), and *O. faveolata* (*p_adj* = 0.022). All other comparisons among AH tissue collected in the epidemic zone were not significant.

### 3.4. Relative Abundance Analysis of Bacterial Class

**Figure 7:**
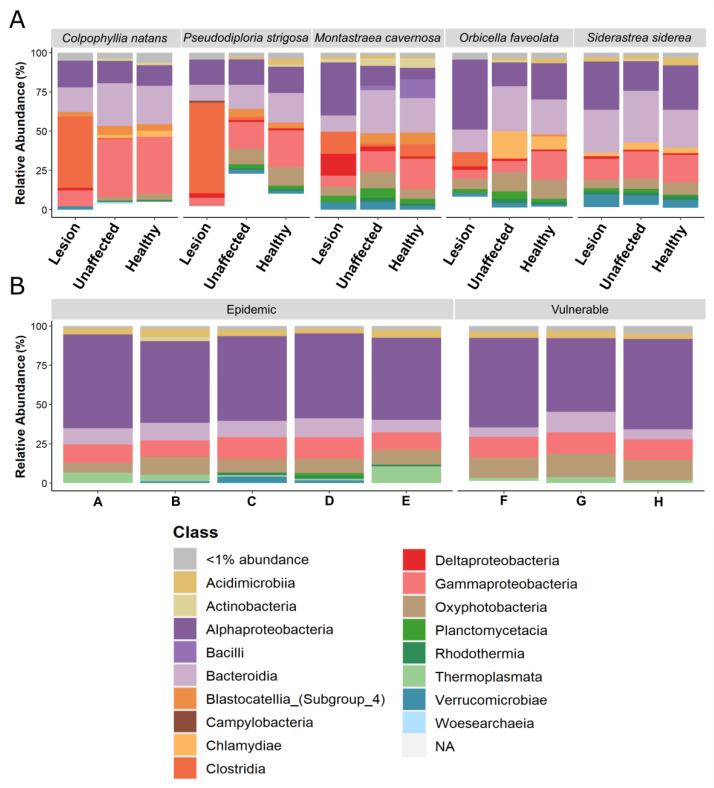
Mean relative abundances of amplicon sequence variants (ASVs) visualized at the bacterial class level in (**A**) lesion and unaffected tissues from colonies with stony coral tissue loss disease, and tissue of apparently healthy colonies, collected from *Colpophyllia natans*, *Pseudodiploria strigosa*, *Montastraea cavernosa*, *Orbicella faveolata*, and *Siderastrea siderea* in the epidemic zone. Additionally, shown are the mean relative abundances of bacterial classes in (**B**) water samples collected across the epidemic zone (Sites A–E) and vulnerable zone (Sites F–H). Displayed are taxa with ≥100 ASV counts in at least 10% of samples. Taxa with <100 ASV counts are represented by the white space.

The relative abundance of bacterial classes revealed interesting patterns with coral species. Of the highly susceptible species *C. natans* and *P. strigosa*, Clostridia was the most dominant bacterial class in the DL tissue (45.11% and 56.22%, respectively; Figure 7A). Following Clostridia, Alphaproteobacteria (16.57%) and Bacteroidia (16.36%) were most abundant for *C. natans* DL tissue. Gammaproteobacteria constituted the highest relative abundance in DU (34.18%) and AH (33.26%) tissues of *C. natans*. In the DU and AH tissues of *P. strigosa*, however, Alphaproteobacteria, Bacteroidia, and Gammaproteobacteria were similarly abundant.

Among the moderately susceptible species, Alphaproteobacteria was generally more dominant in DL tissues. Alphaproteobacteria was the dominant member (31.08%) of DL tissue of *M. cavernosa* even though they were also present, albeit at lower abundances (<13.86%), in AH and DU tissues. These two tissue sample types of *M. cavernosa* had relatively high levels of Bacteroidia (19.02% and 23.28%, respectively) compared to DL tissue (10.49%). In all three *O. faveolata* tissue sample types, Alphaproteobacteria, Bacteroidia, and Oxyphotobacteria were among the highest relatively abundant taxa. While Alphaproteobacteria was ubiquitous across all tissue sample types of *O. faveolata*, this class was more abundant in DL tissue (42.56%) compared to AH and DU tissues (27.17% and 15.32%, respectively). Conversely in *O. faveolata*, AH and DU tissues had higher levels of Bacteroidia (30.54% and 29.09%, respectively) than DL tissue (13.21%). Relatively high abundances of Chlamydia were also present in AH and DU tissues (17.63% and 11.1%, respectively) but not in DL tissue (<1%) of *O. faveolata*. Though not at the same relative abundance levels as in *C. natans* and *P. strigosa*, Clostridia was more abundant (8.6%) in DL tissue than AH and DU tissues (<1%) of *O. faveolata*. Finally, for *S. siderea*, Alphaproteobacteria, Bacteroidia, and Gammaproteobacteria were the most abundant groups of bacteria across all tissue sample types.

At every site in the epidemic and vulnerable zones, the bacterial community of the water column was dominated by Alphaproteobacteria, followed by Gammaproteobacteria, Bacteroidia, and Oxyphotobacteria (Figure 7B).

### 3.5. Analysis of Composition of Microbiomes

Significantly differentially abundant ASVs were detected among the three tissue sample types in each coral species: *C. natans* (33 ASVs with W ≥ 300), *P. strigosa* (88 ASVs with W ≥ 530), *M. cavernosa* (10 ASVs with W ≥ 239), *O. faveolata* (32 ASVs with W ≥ 599), and *S. siderea* (81 ASVs with W ≥ 1524). Sixty-seven significantly differentially abundant ASVs (with W ≥ 557) were detected in the water samples when comparing vulnerable and epidemic zones.

**Figure 8:**
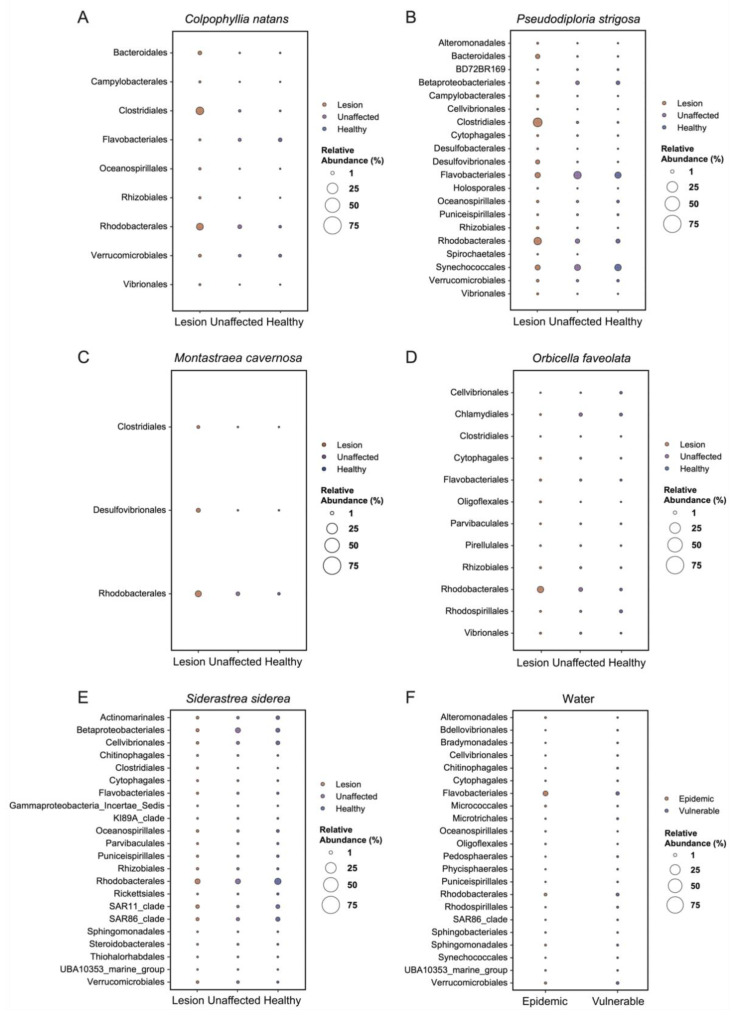
Relative abundances of significantly differentiated taxa (i.e., taxa with W-statistic values greater than a detection threshold of 0.7) in lesion and unaffected tissues from colonies with stony coral tissue loss disease, and tissue of apparently healthy colonies, collected from five coral species: (**A**) *Colpophyllia natans*, (**B**) *Pseudodiploria strigosa*, (**C**) *Montastraea cavernosa*, (**D**) *Orbicella faveolata*, and (**E**) *Siderastrea siderea*. Additionally, displayed is the relative abundance of significant taxa detected in (**F**) water samples collected at sites in the epidemic zone and at sites in the vulnerable zone. Taxa are grouped by order.

The order Rhodobacterales was consistently present at high relative abundances in DL tissue compared to AH and DU tissues for all coral species except *S. siderea* (Figure 8A–E). Eight significantly differentiated Rhodobacterales ASVs were detected in the DL tissue of at least two of the five species of coral: ASV6, ASV21, ASV22, ASV57, ASV116, ASV124, ASV148, and ASV208 (Table 2); all eight of these sequences have been reported in previous studies of SCTLD [39,40,51,52]. ASV148, an unclassified Rhodobacteraceae, was the only ASV detected in the lesions of all five coral species examined here.

The order Clostridiales was also consistently found in the bacterial community of disease tissues. The DL tissues of *C. natans*, *P. strigosa*, and *M. cavernosa* had higher abundances of significantly differentiated Clostridiales compared to AH and DU tissues. ASV50, a *Tepidibacter* belonging to the order Clostridiales, was enriched in the DL tissues of these three coral species. In addition, the abundance of ASV11, a *Halodesulfovibrio* belonging to order Desulfovibrionales, was more abundant in the DL tissue of *M. cavernosa* and *P. strigosa* compared to AH and DU tissues. Water samples collected in the epidemic zone were enriched in Flavobacteriales compared to the vulnerable zone (Figure 8F).

## 4. Discussion

The stony coral tissue loss disease (SCTLD) outbreak has caused the widespread mortality of important reef-building species in Florida and in the Caribbean Region. To date, the identity of the presumed pathogen(s) responsible for SCTLD remains unknown, despite many studies on microbial communities associated with SCTLD [39,40,51,52]. In the present study, the microbiomes of five coral species (*Colpophyllia natans*, *Pseudodiploria strigosa*, *Montastraea cavernosa*, *Orbicella faveolata*, and *Siderastrea siderea*) were analyzed to determine if a specific microbial signature exists across different species of corals with active SCTLD lesions. The lesions of four of these five coral species (except *S. siderea*) had significantly higher abundances of Rhodobacterales, a finding that is consistent with studies of other susceptible coral species (Table 2). In addition to Rhodobacterales, Clostridiales was also a significant and ubiquitous member of the lesion microbial community, especially for *C. natans*, *M. cavernosa*, and *P. strigosa*.

### 4.1. Diversity Indices

In general, the microbial beta diversity dispersion between zones (i.e., vulnerable and epidemic) and within zones was not significant in either water or apparently healthy (AH) coral samples, except for those from *P. strigosa*, which were more dispersed in the vulnerable zone than the epidemic zone. These results, with the exception of *P. strigosa*, are similar to Rosales et al. [40], who found no difference in the AH coral microbial beta diversity dispersion between the vulnerable and epidemic zones. Coral microbiomes are sensitive to environmental perturbations (e.g., thermal stress, nutrient pollution), which can cause an increase in the microbial beta diversity dispersion [53]. It is possible that AH *P. strigosa* colonies in the vulnerable zone had recently been exposed to an environmental stressor, which caused an increase in the beta diversity dispersion. However, longitudinal studies are needed to parse out the influence of environmental conditions on beta diversity dispersion through time. 

The present study did not find a difference in the dispersion of microbial communities in the water column between zones. In Rosales et al. [40], the microbial beta diversity dispersion of water samples was significantly different between zones, with greater dispersion observed within the epidemic zone compared to the vulnerable zone. Even though the beta diversity dispersion of environmental samples (i.e., water samples) did not differ between zones in the present study, differences in environmental conditions between zones should not be ruled out as a possible factor in driving these differences. Additionally, the water sample sizes in the present study were lower than those used in Rosales et al. [40], potentially limiting the ability to observe meaningful differences between the zones in this study. This discrepancy between findings may also be attributed to differences in how the water samples were gathered: in the present study, water sample bottles were held approximately 0.5 m above the benthos and were not specifically gathered above coral colonies. In Rosales et al. [40], water sample bottles were held directly over the benthos, gathering water approximately 20 cm or less from the bottom, and in the epidemic zone, water samples were collected directly over colonies with SCTLD. The distance at which water samples are collected above the bottom influences the microbial signature of the water samples, as illustrated by Weber et al. [54]. Water samples collected within the coral ecosphere, or the environment immediately surrounding an individual coral colony (e.g., water < 30 cm above the colony), can have a different microbial signature compared to water collected >1 m above the reef. Therefore, bacterial beta diversity dispersion may be related to the sampling location (i.e., distance above the benthos or individual coral colonies) in the water column. 

Among the tissue sample types of most coral species, the epidemic zone had a dispersed microbial community. While dispersion was not significantly different among the three tissue sample types in *P. strigosa*, as previously identified in *Diploria labyrinthiformis*, *Dichocoenia stokesii*, and *Meandrina meandrites* [40], the lesion (DL) tissue of the other coral species in this study were more dispersed than AH (in four coral species) and unaffected (DU) tissue (in three coral species, except *C. natans*) tissues. In Meyer et al. [39], microbial dispersion was similar between the DL tissue and DU tissue of diseased colonies from three susceptible species, *Montastraea cavernosa*, *Diploria labyrinthiformis*, and *Dichocoenia stokesii*. However, in *M. cavernosa*, the DL and DU tissues did have a higher dispersion compared to AH tissue. Similarly, in the present study, there were no significant differences between the DL tissue and the DU tissue of *C. natans*. Therefore, colonies showing signs of SCTLD may have a disrupted microbiome even far away from the lesion, suggesting a systemic effect [55]. It has been reported in other studies that stress, such as heat stress, often has a stochastic effect on the microbial community composition that can result in an increase in the beta diversity [56]. In Rosales et al. [57], for example, *Acropora cervicornis* exposed to diseased ramets had a higher beta diversity compared to control corals not exposed to disease. Eaton et al. [55] also showed that visibly unaffected areas (DU tissue) on diseased corals later showed signs of tissue loss after coral fragments were separated and isolated from the active disease border on the parent colony, and Landsberg et al. [30] found lytic necrosis characteristic of SCTLD lesions within some samples of DU tissue, again suggesting that SCTLD may be systemic within coral colonies.

In the epidemic zone, there were also significant differences in the species richness among tissue sample types, with DL tissues having a higher species richness compared to both DU and AH tissues in three species (*C. natans*, *M. cavernosa*, and *S. siderea*) and compared to the AH tissue of two species (*O. faveolata* and *P. strigosa*). This difference in alpha diversity may be a result of microbial dysbiosis, or an imbalance in the natural microbiome that can disrupt coral–microbe interactions and lead to disease [8,58]. The microbiomes of corals exposed to stressful environmental conditions (e.g., acidification, and increased temperature) often experience a shift in microbial community composition and, consequently, an increase in the species richness [8,59]. Microbial shifts may be attributed to a loss in beneficial bacteria; thus, freeing up niche space for putative pathogens to inhabit [13,60]. In a study conducted by MacKnight et al. [58], disease-resistant corals exposed to white plague disease (WPD) had a higher dysbiosis threshold compared to corals that developed WPD lesions. The authors hypothesized that certain bacteria may be helping to prevent pathogens from colonizing disease-resistant corals; thus, also preventing dysbiosis and the onset of WPD. In the present study, the higher microbial alpha diversity of coral DL tissues was potentially due to the decreased stability of the coral microbiomes and the hosts’ inability to prevent pathogenic infection [59], or due to an increased propensity for diseased tissues and surface mucus to become colonized by diverse opportunistic bacteria, including Rhodobacteraceae [61,62].

### 4.2. Differences Were Detected among Sites and Tissue Sample Types

Bacterial communities of all but one species (*O. faveolata*) were different among at least three sites of the epidemic zone (Table S1). Even though sites within the epidemic zone were similarly dispersed, there were significant groupings among sites, suggesting a site-level effect on the bacterial signature of corals within the epidemic zone. There are likely several factors driving these site-wide differences. In a previous study, Williams et al. [34] showed that the coral species diversity, coral cover, and size of coral colonies affect SCTLD prevalence and severity. Sites with higher abundances of *M. cavernosa* and *O. faveolata* compared to four other susceptible species (*C. natans*, *P. strigosa*, *Diploria labyrinthiformis*, and *Dichocoenia stokesii*) had a greater disease prevalence. In addition, colonies that ultimately became diseased were significantly larger than colonies that did not display signs of SCTLD over the course of the study. This finding was also observed by Sharp et al. [63]. Both studies [34,63] observed that the coral density did not likely play a role in the spatio-temporal dynamics of SCTLD. While coral density may not be a factor, differences in the coral species diversity and size of colonies among sites in the present study may explain the site-level differences observed in microbial communities. Even though the microbial communities of these corals are site-specific (Table S1), there were consistent signatures within the DL tissues of corals among sites.

### 4.3. Rhodobacterales and Clostridiales in SCTLD Lesions

The DL tissues of four species in this study (*C. natans*, *P. strigosa*, *O. faveolata*, and *M. cavernosa*) had significantly higher relative abundances of Rhodobacterales compared to both DU and AH tissues. In previous studies, Rhodobacterales was also differentiated in the lesions of *Stephanocoenia intersepta*, *Diploria labyrinthiformis*, *Dichocoenia stokesii*, *Meandrina meandrites,* and *Montastraea cavernosa* in Florida [39,40], and, although not significant, Rhodobacteraceae was enriched in the lesion tissues of *Meandrina meandrites* and *O. franksi* in the U.S. Virgin Islands [52]. Other studies have reported high abundances of Rhodobacterales, specifically *Rhodobacter*, in disease tissue of black band disease, white plague, and white band disease [64,65,66,67,68,69]. This group of bacteria play an important role in colonizing submerged marine surfaces and is often considered the primary and most common colonizer [70,71]. The fast-growing nature of Rhodobacterales allows members of this group to thrive in areas that are rich in amino acids and other nutrients [72]. These bacteria also have the ability to produce antibiotic compounds [73]. Under stressful conditions, Rhodobacterales appear to be typical and abundant opportunistic bacteria associated with corals [61,62,74].

In this study, an exception to the pattern of higher relative abundance of Rhodobacterales in DL compared to both DU and AH tissues was the coral *S. siderea*. The DU tissues of *S. siderea* had a lower abundance of Rhodobacterales compared to both DL and AH tissues, but the latter tissue sample types had statistically similar abundances. *Siderastrea siderea* often shows signs of SCTLD that are distinct from other susceptible species, including areas of pinkish tissue discoloration and mucus strands [30], leading to some speculation about whether this species has the same disease or a generalized stress response [25]. However, the characteristic hallmark lytic necrosis of SCTLD as described in the lesion tissues of other susceptible species has been reported in *S. siderea* tissue [30], so differences in how the tissue loss progresses through the colony in *S. siderea* (generally originating in polyp mouths instead of moving across the colony [30]) or its species-specific holobiont response may affect the gross presentation of the disease and the relative abundances of bacteria across tissue sample types compared with other species. However, it should be noted that the method of sampling for *S. siderea* (predominantly coring and scraping versus scraping only) may have influenced the composition of the microbial community in the samples examined, especially if less tissue and mucus were potentially sampled. Surface mucus on corals has a diverse microbial community, the composition of which changes during the course of disease [61]. Scraping with a coring device may have obtained slightly deeper tissue samples which may have a different microbial composition. Studies to evaluate the vertical and horizontal distribution and abundances of the microbial flora in SCTLD-affected colonies over time in relation to lesion progression, coral species, sampling method, and mucus quantity may resolve this question.

In the present study, Clostridiales was another significantly differentiated group of bacteria in lesion tissue. ASV50 specifically was enriched in the lesion tissue of *C. natans*, *M. cavernosa*, and *P. strigosa*. Of these corals, *C. natans* and *P. strigosa* are highly susceptible to SCTLD, and exhibit acute to subacute tissue loss [25,30]; *M. cavernosa* is considered moderately susceptible, but the rate of lesion progression varies widely by colony, and most of the diseased colonies sampled in this study were experiencing subacute tissue loss (authors’ pers. obs.). It is possible that Clostridiales is a signature of faster lesion progression, given that this order has been documented in other studies in *M. cavernosa* and *C. natans* [39,52] in addition to two other highly susceptible species: *Dichocoenia stokesii* and *Diploria labyrinthiformis* [39]. However, Rosales et al. [40] did not observe significantly differentiated Clostridiales in DL tissue of *Dichocoenia stokesii* or *Diploria labyrinthiformis*, but only within DL tissues of *Stephanocoenia intersepta*, another moderately susceptible species [25]. Similar to Rhodobacterales, Clostridiales is commonly found in the lesion tissue of other coral diseases, such as black band disease, white plague disease, and white syndrome [67,68,75]. When colonizing, these opportunistic anaerobic bacteria can necrotize host tissue. For example, humans and animals exposed to clostridial spores (e.g., through contaminated drinking water) can contract clostridial myonecrosis (gas gangrene) which is a lethal infection that causes severe necrosis of muscle and soft tissue [76]. The initial appearance of SCTLD lesions in deeper basal body wall tissues may indicate a role for anaerobic bacterial pathogenesis, although thus far, no histological evidence has been found for co-occurring bacteria in lesion initiation [30]. However, the exact role, if any, of Clostridiales in lesion progression and tissue necrosis of coral colonies with SCTLD, should be examined among highly susceptible species.

## 5. Conclusions

The goal of this study was to determine if the lesions of coral colonies showing signs of stony coral tissue loss disease (SCTLD) had a consistent microbial signature across five different coral species: *Colpophyllia natans*, *Pseudodiploria strigosa*, *Montastraea cavernosa*, *Orbicella faveolata*, and *Siderastrea siderea*. Diversity indices revealed that the lesion (DL) tissue of all but one species (*P. strigosa*) had a higher microbial beta diversity dispersion than apparently healthy (AH) tissue. In three species (*M. cavernosa*, *O. faveolata*, and *S. siderea*), DL tissue had a higher beta diversity dispersion than both AH tissue and unaffected (DU) tissue. Furthermore, DL tissue of every species had a higher species richness compared to AH tissue. In three species (*C. natans*, *M. cavernosa*, and *S. siderea*), the species richness of DL tissue was also higher than DU tissue. DL tissue consistently had higher relative abundances of the order Rhodobacterales compared to AH and DU tissues, except for *S. siderea*, a finding that has been observed in other coral species [39,40,51,52]. In addition, order Clostridiales was enriched in the DL tissue of three of the five species investigated in the present study, suggesting that Clostridiales may also play an important role in SCTLD. The beta diversity dispersion and species richness of water samples did not differ between the vulnerable and epidemic zones, suggesting that a bacterial signature of SCTLD was not detected in the water column, potentially due to sampling location within the sites. The results presented herein expand our understanding of SCTLD as it relates to the coral microbiome of critical reef-building species along Florida’s Coral Reef. Understanding how SCTLD, coupled with changing environmental conditions, can affect host-microbe interactions is an important step towards developing practical and effective disease mitigation strategies.

## Figures and Tables

**Table 1:** Summary of coral tissue/mucus and water samples collected from sites (n = 8; Figure 1) along Florida’s Coral Reef. Shown are the dates of sampling, stony coral tissue loss disease (SCTLD) zone, site letter codes, and coral species sampled: *Colpophyllia natans* (CNAT), *Pseudodiploria strigosa* (PSTR), *Montastraea cavernosa* (MCAV), *Orbicella faveolata* (OFAV), and *Siderastrea siderea* (SSID). For each coral species, the number of lesion and unaffected tissue samples from colonies with SCTLD, and the number of samples from apparently healthy colonies, collected per site are shown. The number of water samples collected per site is also shown.

Date	Zone	Site Letter Code ^1^	Coral Species	Lesion/ Site	Unaffected/ Site	Healthy/ Site	Water/ Site
04/09–04/27/18	Epidemic	A–E	CNAT	5	5	3	-
04/09–04/27/18	Epidemic	A–E	PSTR	4–5	4–5	3	-
04/09–04/27/18	Epidemic	A–E	MCAV	5	5	3	-
04/09–04/27/18	Epidemic	A–E	OFAV	3–5	3–5	3	-
04/09–04/27/18	Epidemic	A–E	SSID	5	5	3	-
04/09–04/27/18	Epidemic	A–E	-	-	-	-	3
05/08–06/05/18	Vulnerable	F–H	CNAT	-	-	3	-
05/08–06/05/18	Vulnerable	F–H	PSTR	-	-	2–3	-
05/08–06/05/18	Vulnerable	F–H	MCAV	-	-	3	-
05/08–06/05/18	Vulnerable	F–H	OFAV	-	-	3	-
05/08–06/05/18	Vulnerable	F–H	SSID	-	-	3	-
05/08–06/05/18	Vulnerable	F–H	-	-	-	-	3
Total				122	122	119	24

^1^ Site: A—West Turtle Shoal; B—Boot Key Patch; C—Nearshore Patch; D—East Turtle Shoal; E—Dustan Rocks; F—Western Sambo Patch; G—Xesto Patch; H—Lindsay’s Patch.

**Table 2:** Significantly differentiated amplicon sequence variants (ASVs) enriched in the lesion tissue of ≥2 of the coral species examined in this study: *Colpophyllia natans*, *Pseudodiploria strigosa*, *Montastraea cavernosa*, *Orbicella faveolata*, and *Siderastrea siderea*. NCBI’s Basic Local Alignment Search Tool (BLAST) confirmed that the ASVs listed here match sequences published in other stony coral tissue loss disease studies.

ASV ID	Family, Genus	Reference
6	Rhodobacteraceae, *Ruegeria*	[39,51]
21	Rhodobacteraceae, *Thalassobius*	[39,40,52]
22	Rhodobacteraceae, *Shimia*	[39,40,51,52]
50	Peptostreptococcaceae, *Tepidibacter*	[39,51]
57	Rhodobacteraceae, *Nautella*	[39]
116	Rhodobacteraceae, *Planktotalea*	[39,51]
124	Rhodobacteraceae, *Salinihabitans*	[39]
148	Rhodobacteraceae, unclassified	[39]
208	Rhodobacteraceae, *Nioella*	[39,51]

## Data Availability

The 16S rRNA sequence data presented in this study are available through NCBI’s Sequence Read Archive under BioProject number PRJNA772194. The taxa table and amplicon sequence variant (ASV) counts table are publicly available on GitHub at https://github.com/abigailclark1987/sctldmicrobiome.git (accessed on 12 October 2021).

## Supplementary Materials

The following are available online at https://www.mdpi.com/article/10.3390/microorganisms9112181/s1, Table S1: results of permutational multivariate analysis of variance (PERMANOVA) and pairwise PERMANOVA tests comparing microbial communities among sites in the epidemic zone and vulnerable zone, Figure S1: microbial alpha diversity metrics (species richness and Shannon diversity) comparing five corals species between the epidemic zone and vulnerable zone, Figure S2: microbial alpha diversity metrics (species richness and Shannon diversity) comparing five corals species among sites (Sites A–E) in the epidemic zone, Figure S3: microbial alpha diversity metrics (species richness and Shannon diversity) comparing five corals species among sites (Sites F–H) in the vulnerable zone.
