# Supplementary material for: Characterization of the Microbiome of Corals with Stony Coral Tissue Loss Disease along Florida’s Coral Reef
Source: Microorganisms. 2021 Oct 20;9(11):2181. doi: 10.3390/microorganisms9112181 (PMC8623284; doi:10.3390/microorganisms9112181)
Supplement: Supplementary file 1 [file microorganisms-09-02181-s001.zip › microorganisms-1383524 suppl final.pdf]

microorganisms

MDPI

Supplementary Materials

Table S1. Results of permutational multivariate analysis of variance (PERMANOVA) and pairwise PERMANOVA tests comparing microbial communities among sites in the epidemic zone and vulnerable zone. Shown are the stony coral tissue loss disease (SCTLD) zones, site letter codes, and coral species sampled: *Colpophyllia natans* (CNAT), *Pseudodiploria strigosa* (PSTR), *Montastraea cavernosa* (MCAV), *Orbicella faveolata* (OFAV), and *Siderastrea siderea* (SSID). For each coral species, the F statistic (with degrees of freedom, df), R^2, *p*-value, *p*-value adjusted with Bonferroni correction (*p_adj*), and significant sites based on *p_adj* values. All PERMANOVA analyses were performed using a Euclidean distance.

Zone	Site Letter Code [1]	Coral Species	F-test(df)	R^2	*p*-value	*p_adj*	Significant Sites
Epidemic	A–E	CNAT	1.655(4,50)	0.10	<0.00001	<0.03	A,B,D,E
Epidemic	A–E	PSTR	1.4728(4,48)	0.089	<0.00001	<0.04	A vs E; B,D,E
Epidemic	A–E	MCAV	1.3718(4,50)	0.083	0.0002	<0.01	A vs B,C,D
Epidemic	A–E	OFAV	1.3117(4,46)	0.085	0.0015	>0.10	None
Epidemic	A–E	SSID	1.7462(4,50)	0.10	0.00001	<0.02	A vs B,C,D,E<br>B vs D,E
Vulnerable	F–H	CNAT	1.0744(2,6)	0.26	0.27	-	None
Vulnerable	F–H	PSTR	1.2867(2,5)	0.34	0.057	-	None
Vulnerable	F–H	MCAV	1.2662(2,6)	0.30	0.0038	0.30	None
Vulnerable	F–H	OFAV	1.5025(2,6)	0.33	0.0037	0.30	None
Vulnerable	F–H	SSID	1.2822(2,6)	0.30	0.0072	0.30	None

[1] Site: A—West Turtle Shoal; B—Boot Key Patch; C—Nearshore Patch; D—East Turtle Shoal; E—Dustan Rocks; F—Western Sambo Patch; G—Xesto Patch; H—Lindsay's Patch.

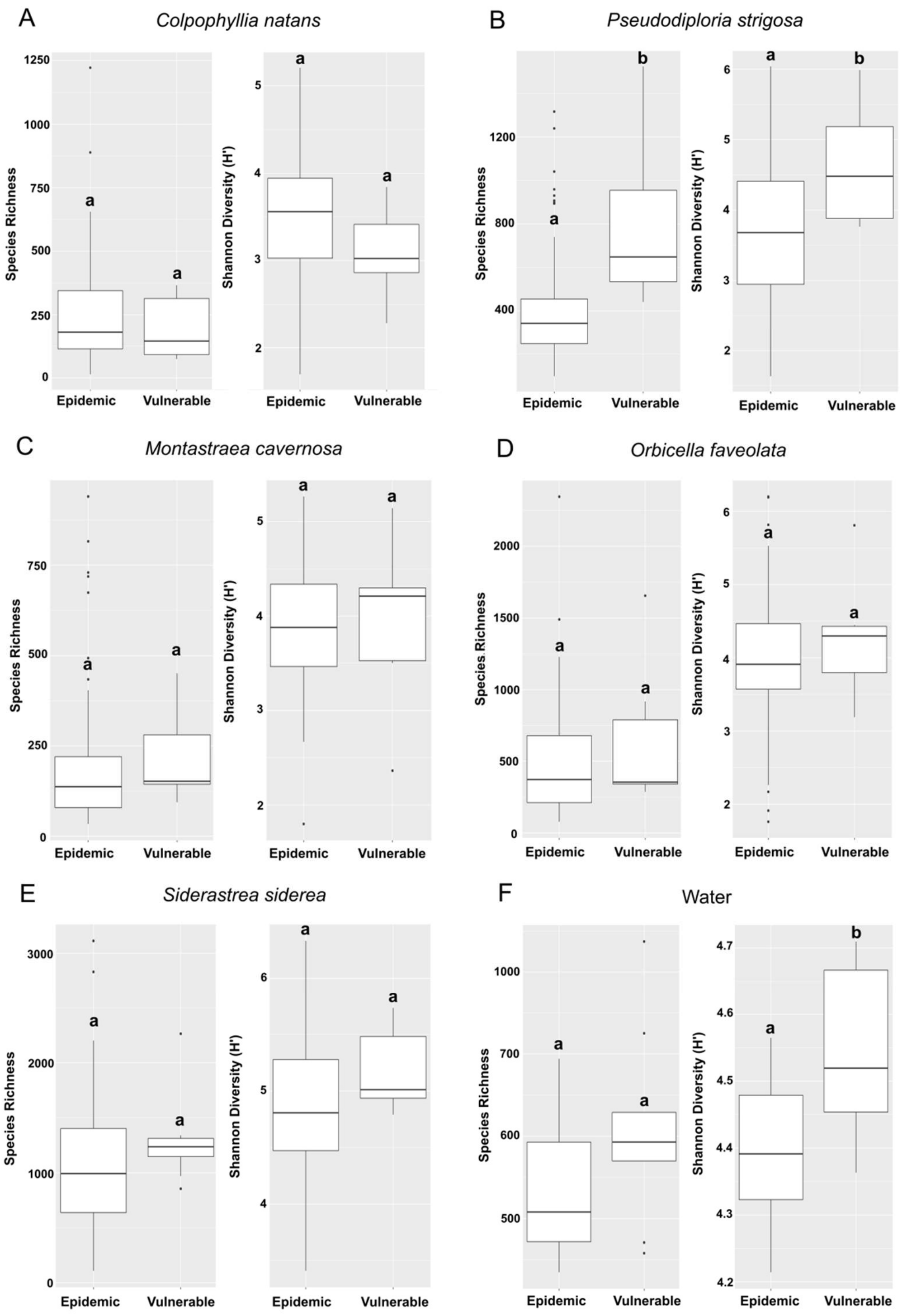

Figure S1. Microbial alpha diversity metrics (species richness and Shannon diversity) comparing five corals species between the epidemic zone and vulnerable zone: (**A**) *Colpophyllia natans,* (**B**) *Pseudodiploria strigosa,* (**C**) *Montastraea cavernosa,* (**D**) *Orbicella faveolata,* and (**E**) *Siderastrea siderea.* For the epidemic zone, all three tissue types (the lesion tissue and the unaffected tissue of diseased coral colonies, and tissue from apparently healthy coral colonies) were pooled and compared with the tissue from apparently healthy coral colonies in the vulnerable zone. Also displayed are microbial alpha diversity metrics of (**F**) water samples from each zone. Letters denote significant differences determined by the post-hoc tests.

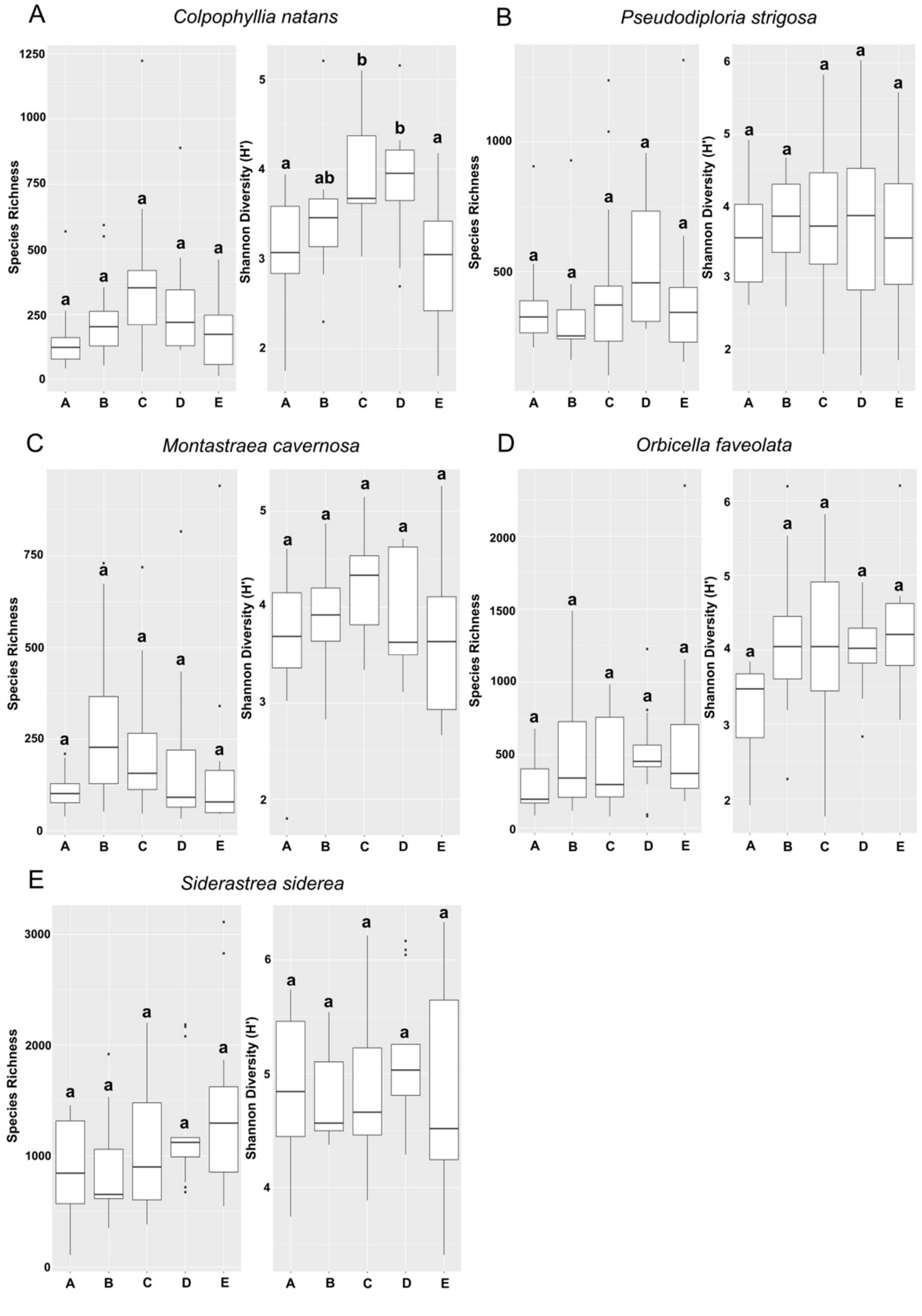

Figure S2. Microbial alpha diversity metrics (species richness and Shannon diversity) comparing five corals species among sites (Sites A–E) in the epidemic zone: (**A**) *Colpophyllia natans*, (**B**) *Pseudodiploria strigosa*, (**C**) *Montastraea cavernosa*, (**D**) *Orbicella faveolata*, and (**E**) *Siderastrea siderea*. Diversity metrics are from all sampled tissue types pooled (lesion and unaffected tissue from diseased colonies and apparently healthy colony tissue). Letters denote significant differences determined by the post-hoc tests.

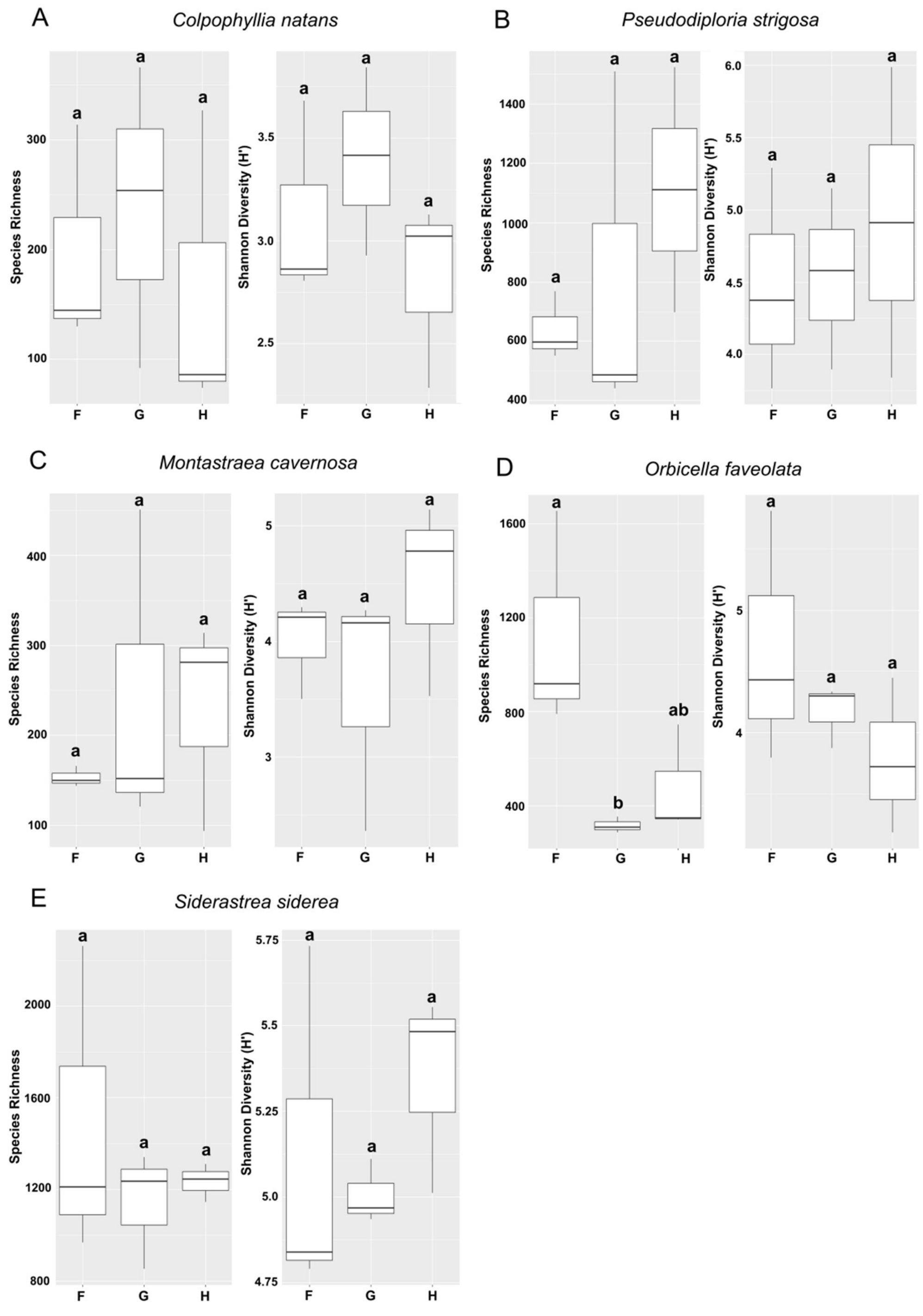

Figure S3. Microbial alpha diversity metrics (species richness and Shannon diversity) comparing apparently healthy colony tissue from five corals species among sites (Sites F–H) in the vulnerable zone: (**A**) *Colpophyllia natans,* (**B**) *Pseudodiploria strigosa,* (**C**) *Montastraea cavernosa,* (**D**) *Orbicella faveolata,* and (**E**) *Siderastrea siderea.* Letters denote significant differences determined by the post-hoc tests.
